# Cystic echinococcosis in Iceland: a brief history and genetic analysis of a 46-year-old *Echinococcus* isolate collected prior to the eradication of this zoonotic disease

**DOI:** 10.1017/S0031182023000355

**Published:** 2023-06

**Authors:** Urmas Saarma, Karl Skirnisson, Thorunn Soley Björnsdottir, Teivi Laurimäe, Liina Kinkar

**Affiliations:** 1 Department of Zoology, Institute of Ecology and Earth Sciences, University of Tartu, J. Liivi 2, 50409 Tartu, Estonia; 2 Laboratory of Parasitology, Institute for Experimental Pathology, University of Iceland, Keldur, Reykjavik, Iceland; 3 PCR Laboratory, Institute for Experimental Pathology, University of Iceland, Keldur, Reykjavik, Iceland

**Keywords:** Cystic echinococcosis, *Echinococcus granulosus sensu stricto*, genotype G1, Iceland, mitochondrial DNA

## Abstract

Cystic echinococcosis (CE) is considered the most severe parasitic disease that ever affected the human population in Iceland. Before the start of eradication campaign in the 1860s, Iceland was a country with very high prevalence of human CE, with approximately every fifth person infected. Eradication of CE from Iceland by 1979 was a huge success story and served as a leading example for other countries on how to combat such a severe One Health problem. However, there is no genetic information on *Echinococcus* parasites before eradication. Here, we reveal the genetic identity for one of the last *Echinococcus* isolates in Iceland, obtained from a sheep 46 years ago (1977). We sequenced a large portion of the mitochondrial genome (8141 bp) and identified the isolate as *Echinococcus granulosus sensu stricto* genotype G1. As G1 is known to be highly infective genotype to humans, it may partly explain why such a large proportion of human population in Iceland was infected at a time . The study demonstrates that decades-old samples hold significant potential to uncover genetic identities of parasites in the past.

## Graphical abstract

**Figure:**
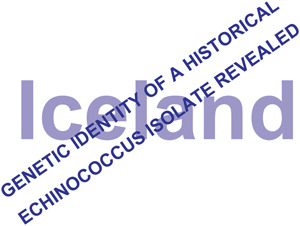

## Introduction

Cystic echinococcosis (CE) is a severe parasitic disease affecting both humans and animals (Jenkins *et al*., 2015; Deplazes *et al*., 2017; Kern *et al*., 2017). CE is caused by a complex of tapeworm species collectively referred to as *Echinococcus granulosus sensu lato* (*s.l.*). These include the species *E. granulosus sensu stricto* (*s.s*.; genotypes G1 and G3; Kinkar *et al*., 2017), *E. equinus* (G4), *E. felidis*, *E. ortleppi* (G5) and the *E. canadensis* cluster (G6–G8, G10) for which the number and name(s) of species are under dispute (e.g. Moks *et al*., 2008; Thompson, 2008; Saarma *et al*., 2009; Knapp *et al*., 2011; Nakao *et al*., 2015; Romig *et al*., 2017; Laurimäe *et al*., 2018*a*; Vuitton *et al*., 2020). The life cycle of these tapeworms involves canids as definitive hosts, harbouring the adult worm, and various herbi- and omnivores that act as intermediate hosts. In the latter, the larval stage develops predominantly in the liver or lungs in the form of fluid-filled cysts (Thompson, 2017). Several *E. granulosus s.l.* species are transmitted mostly between domesticated animals, with dogs acting as the main definitive and livestock ungulates as intermediate hosts, whereas others infect largely wildlife species, such as wolves and moose (Romig *et al*., 2017). Humans are accidental aberrant hosts and do not generally contribute to the perpetuation of the life cycle of the parasite (Deplazes *et al*., 2017). A systematic review, based on European *E. granulosus s.l.* data published in 2000–2021, identified 599 genetically confirmed human echinococcal cysts, majority of which belonged to *E. granulosus s.s.* (76.8%), followed by genotypes G6/7 and G10 of the *E. canadensis* cluster (21.7%) (Casulli *et al*., 2022). However, the number of human CE cases is likely an underestimation. A recent Europe-wide analysis has revealed that the disease is under-reported (Casulli *et al*., 2023). The most abundant species – *E. granulosus s.s.* – is predominantly transmitted among domesticated animals (e.g. sheep and dogs) (Romig *et al*., 2017).

### A brief historical review of CE in Iceland

CE ('sullaveiki’ in Icelandic) was likely introduced to Iceland during the settlement period in the 9th century and was considered endemic already in the 12th–13th centuries (mentioned in Icelandic sagas written in this period) (Skírnisson *et al*., 2003 and references therein; Kristjansdottir and Collins, 2011). In addition to these documents, there is also archaeological evidence of CE in humans in Iceland in the past. A cyst as large as 15 cm in diameter was found in a female skeleton, aged about 45 years, from a cemetery dated to the 18th century at Viðey in Southwest Iceland (Gestsdottir, 2004). Interestingly, the most ‘prolific’ site has been Skriðuklaustur, a medieval Augustinian monastery in eastern Iceland that also functioned as a hospital in 1493–1554. In this region, Zoega (2007) reported of a skeleton diagnosed with ‘hydatism’. Later on, 8 additional cases were recovered by Kristjansdottir and Collins (2011), including a skeleton with large cysts of 17–20 cm in diameter. *Echinococcus* cysts in archaeological material have also been reported in other parts of Europe, such as Denmark (Weiss and Møller-Christensen, 1971).

Reports dating back to the 19th century indicate that CE had devastating health consequences in Iceland during this time (Fig. S1), and according to a local medical doctor, nearly every fifth adult died of CE or its complications (Hjaltelin, 1869). In the 19th century and in the first half of the 20th century, Iceland had the highest prevalence of human CE ever recorded anywhere (Dungal, 1957). For example, of 2272 autopsies carried out in Reykjavik (during 1932–1950), CE prevalence was documented to range between 15 and 22% among people born between 1841 and 1880. The infection rate in humans was likely high due to effective transmission of the parasite between sheep and dogs. Slaughtering occurred in farms and offal were often fed to dogs. In the mid-19th century, approximately 70 000 humans inhabited Iceland, alongside with 600 000–700 000 sheep and 15 000–20 000 dogs. Systematic data on the prevalence of CE in animals in Iceland are scarce and the information is scattered. Jón Finsen in his medical reports from 1858 considered ‘hydatids’ (i.e. *Echinococcus* cysts) so common in ‘kvíaám’ (ewes being milked without lambs) and cattle, that animals free of infection were an exception. Thus, almost all sheep and cattle were infected when slaughtered (Pálsson *et al*., 1953). Also, Krabbe (1865) reported that *Echinococcus* was very common in sheep and cattle, and was found also in a pig. In 1863, 28% of dogs were reported to be infected (Krabbe, 1865).

### Eradication efforts of CE in Iceland

Measures to control CE were already formulated in the 1860s to reduce parasite transmission and included preventing dogs from accessing raw offal, restricting home slaughter of sheep, improved meat inspection and appropriate offal disposal and educating people about hygiene and the life cycle of the parasite. These measures were first applied in 1863, whereas in 1869 the first nationwide legislation to control CE was enforced to regulate the number of dogs and placing a tax on all dogs not required for work. The law also stipulated the instructions for appropriate destruction of cysts and infected offal (Beard, 1973). For deworming, the kamala fruit (*Mallotus philippinensis*) extract was used in the beginning, although this was soon replaced with a more effective areca nut (*Areca catechu*) extract containing arecoline as a key ingredient. From 1924, a synthetic arecoline hydrobromide was used (reviewed in Craig *et al*., 2017). This drug caused diarrhoea and did not eliminate all cestodes from the organism. Eradication efforts of CE in Iceland were further strengthened by changes in sheep husbandry, favouring exporting and marketing wethers, decreasing thereby the rates of home slaughter. As a result, human CE cases were reduced significantly.

Measures to combat CE taken since 1860s in Iceland proved to be highly successful, resulting in the disease being virtually eliminated by 1920 (prevalence in the human population between 1881 and 1920 ranging from 0 to 3%; Dungal, 1957) and only 8 cases being reported in the 20th century (Skírnisson *et al*., 2003; Skírnisson, 2017). Four of these cases were diagnosed in 1984–1988 (Arinbjarnar, 1989; Fig. S2). These patients had likely acquired the infection in the first half or middle of the century. The last mortality case was in 1960, when a 23-year-old woman died of complications during surgery (Jónsson, 1962). The same trend occurred in livestock animals. In 1924 there was a survey in slaughter houses, where 17 424 sheep were examined and 2164 had hydatids (12.4%), 763 in the liver, 15 in the lungs and 75 both in the liver and lungs. The prevalence decreased in the following decades. By the middle of the 20th century, CE in sheep and cattle had more or less disappeared. The last infected cattle was recorded in 1961 and sheep in 1979 (Pálsson *et al*., 1953; Pálsson, 1984; Skírnisson, 2017). The cestode survived regionally in dogs in the eastern part of the country until 1970s (Pálsson, 1984; Skírnisson, 2017). It is worth noting that no anthelmintic effective against cestodes was used in Iceland until praziquantel was adopted in the 1970s. Although several factors contributed to the gradual eradication of CE, education on the disease and prevention, as well as decreased onsite slaughter of adult sheep coupled with restricting access of dogs to raw offal, played a major role (Beard, 1973). Thus, the control programme to eliminate CE in Iceland was highly successful and has served as an example for other countries faced with similar challenges. In the middle of the 20th century, CE control programmes were initiated in other islands such as New Zealand, Tasmania, Cyprus and Falkland Islands (Malvines), eventually resulting in the eradication of the disease (Craig *et al*., 2017).

To date, no *Echinococcus* cyst material from Iceland has been molecularly characterized. To understand which species/genotypes of *E. granulosus s.l.* were present in Iceland before the eradication of CE, we aimed here to sequence the coding regions of the mitochondrial genome of *Echinococcus* isolates obtained from one of the last known infected sheep from 1977. We also explored their phylogenetic relations with other samples from Europe for which mitochondrial genome sequences are publicly available.

## Material and methods

### Biological material

Three *Echinococcus* cysts, 2 from the liver and 1 from the lungs, were collected in 1977 from an adult female sheep (ID number H3479/77; Óseyri, Stöðvarfjörður, Iceland; Fig. S3). The sheep was the second to last animal found to be infected in Iceland. The affected organs were collected by veterinarian Hákon Hanson during a routine inspection in the slaughterhouse of Breiðdalsvík and sent to the Keldur Institute where a local expert Halldór Vigfússon identified the isolates as ‘*Sullaveiki*’ (i.e. *E. granulosus*). After microscopical examination, he noted the following: ‘*Huge numbers of protoscolices present with typical hooks, the hooks have the typical shape of those of Echinococcus*’ (Fig. S4). Halldór Vigfússon was an experienced scientist who had worked on the identification of *Echinococcus* isolates for decades. In addition to the 3 cysts, more were found in the lungs and liver, both on the surface and deeper inside the organs (most appearing greyish), but were not collected. Calcification was not noticed in any of the cysts. After the examination, the collected samples were stored frozen (−20°C). In 2022, scrapings were taken from the inside of the 3 cysts and placed in 99% ethanol. It is worth noting that the samples had thawed up at least once prior to this.

### Sample preparation, PCR and sequencing

Genomic DNA was extracted from parasite material. The samples were digested in ATL buffer with protease K, and the DNA was extracted using the Qiamp96 DNA Qiacube HT Kit (Qiagen, Germany) following manufacturer's instructions. Before polymerase chain reaction (PCR) amplification, the quality of the DNA was verified by a NanoDrop 2000 spectrophotometer (Thermo Fisher Scientific, Waltham, USA). Three DNA samples, 1 from each cyst, were PCR amplified. PCR reactions were set up and amplification was performed as described in Laurimäe *et al*. (2018*b*), involving mitogenome amplification with 13 primer pairs and yielding overlapping DNA fragments. PCR reaction was performed in a volume of 20 *μ*L, with 1× BD Advantage 2 PCR buffer (BD Biosciences, Franklin Lakes, NJ, USA), 0.2  *μ*M deoxyribonucleoside triphosphate (Thermo Fisher Scientific), 0.25 *μ*M of each primer, 1 U of Advantage 2 Polymerase mix (BD Biosciences) and 10–100 ng of template DNA. PCR amplification was performed in a touchdown regime: 95°C for 1 min, followed by 10 cycles of 95°C for 20 s, 55°C for 45 s (annealing temperature reduced in each cycle by −0.5°C) and 68°C for 2 min; followed by 25 cycles of 95°C for 20 s, 50°C for 45 s, 68°C for 2 min; and finishing with 68°C for 3 min. Nine microlitres of the PCR products were examined on a 1.0% agarose gel and 10 *μ*L were purified with 1 U shrimp alkaline phosphatase/1 U exonuclease I mix (both from Thermo Fisher Scientific) by incubating at 37°C for 30 min and then inactivating the enzymes at 80°C for 15 min. Sequencing was performed at the Core Facility at the Institute of Genomics (University of Tartu, Estonia), using the same set of primers as for the initial PCR. Both forward and reverse strands were sequenced.

### Sequence assembly, quality control and alignment

Consensus sequences were assembled in Codon Code Aligner v.10.0.2. Chromatograms were checked by eye and mistakes were corrected. Sequence alignment was performed by using Clustal W (Thompson *et al*., 1994), integrated into BioEdit v.7.2.5 (Hall, 1999).

### Phylogenetic analysis

Mitochondrial genome sequences of other *E. granulosus s.s.* G1 isolates from Europe were retrieved from GenBank (Table 1) and aligned with the sequence of this study. To evaluate the phylogenetic position of the Icelandic G1 haplotype among the sequences retrieved from GenBank, a median-joining network was constructed, using Network v.10.1.00 (Fluxus Technology Ltd., Colchester, UK) software, with both indels and point mutations considered.

**Table 1.** A list of haplotypes and the GenBank accession numbers of the corresponding sequences. Abbreviations: ISL – Iceland, SPA – Spain, ITA – Italy, ROM – Romania, MOL – Moldova, ALB – Albania, FRA – France, GRE – Greece.

Haplotype in Figure 1	GenBank accession no.
ISL1*	OQ269593
SPA1	MG672137
SPA2	MG672139
SPA3	MG672147
SPA4	MG672148
SPA5	MG672149
SPA6	MG672150
SPA7	MG672151
SPA8	MG672152
SPA9	MG672153
SPA10	MG672154
SPA11	MG672129
ITA1	MG672133
ITA2	MG672134
ITA3	MG672135
ITA4	MG672136
ITA5	MG672277
ITA6	MG672278
ITA7	MG672279
ITA8	MG672280
ITA9	MG672281
ROM1	MG672131
ROM2	MG672138
MOL1	MG672144
MOL2	MG672145
MOL3	MG672146
ALB1	MG672140
ALB2	MG672141
FRA1	MG672143
GRE1	MG672130
GRE2	MG672282

* Sequenced in this study. The other sequences are from Kinkar *et al.* (2018*b*)

## Results

### Genotypic identity

All 3 DNA samples analysed from different cysts were identified as *E. granulosus s.s.* genotype G1 and were identical in their sequence. However, the DNA samples were not of equal quality, and a single isolate (from lung) that produced the best sequence quality was selected for further analysis. This sequence was of 8141 bp in length, and included the complete *cox1*, *rrnL*, *rrnS*, *cytb*, *nad4L*, *atp6*, *nad2*, *nad1* and *nad3* gene sequences, as well as partial *nad5*, *cox2* and *nad4* gene sequences. The gene sequences were concatenated and deposited to the GenBank database (accession number OQ269593).

### Phylogeographic relations of the Icelandic isolate with other G1 isolates in Europe

The median-joining phylogenetic analysis, based on an alignment of 31 sequences (8141 bp), produced a star-like network, where the Icelandic G1 haplotype was separated from the central putative haplotype (a hypothetical common ancestor of all analysed isolates) by 2 mutations and did not specifically belong to any haplogroup. None of the haplotypes is highly diverged, the closest to the central haplotype is 2 mutational steps apart (the one from Iceland and an isolate from Spain (SPA11). The other haplotypes are 3–11 mutational steps apart from the putative common ancestor. In the network, several groups can be identified, comprising of isolates from: (1) Italy, (2) Italy and Spain, (3) Spain; (4) Romania and Moldova, (5) Albania, Romania and Moldova.

## Discussion

It is likely that the genotype identified in this study (G1) was one of the representative genotypes circulating among humans and livestock in Iceland prior to the eradication of CE, and possibly contributed significantly to the high rates of infection in humans. Although other genotypes may have been present in Iceland that are also known to infect humans, their impact was likely lower, as historic records in Iceland indicate that CE was mainly recorded from sheep, and *E. granulosus s.s.* (i.e. the former ‘sheep strain’; G1) is known to cause the majority of human CE infections in Europe (Casulli *et al*., 2023), as well as globally (Alvarez Rojas *et al*., 2014). As sporadic CE infections were also recorded from cattle and pigs (Pálsson, 1984), we cannot exclude the presence of *E. ortleppi* (G5) and/or *E. granulosus s.l.* genotype G7 in Iceland, though *E. granulosus s.s.* is known to infect these hosts as well (Romig *et al*., 2017). Although other potential *E. granulosus s.l.* hosts – reindeer and horse – were also present before eradication, CE has never been reported from these animals in Iceland (Skírnisson, 2017).

The current study highlights that mitochondrial DNA from *Echinococcus* cyst material that has been stored for several decades at −20°C can be successfully sequenced, despite at least 1 freeze/thaw cycle. Mitochondrial DNA integrity appeared to be relatively high, as the primer pairs used in the current study were designed to amplify approximately 1000–1500 bp. This effort yielded a total sequence length of 8141 bp, which is a relatively large portion of the complete mitochondrial genome of G1 (17 675 bp; Kinkar *et al*., 2019). There are also other reports of DNA analysis from long-term preserved samples of *Echinococcus*. For example, DNA sequencing was successfully performed (and viability demonstrated) for metacestodes of *Echinococcus multilocularis*, cryopreserved for 35 years (Laurimäe *et al*., 2020).

Although one of the main goals here was to identify the species/genotype of the Icelandic *E. granulosus s.l.* isolate, for which analysis of short sequences is sufficient (e.g. the *nad*5 and *nad*2 gene fragments; Kinkar *et al*., 2018*a*; Laurimäe *et al*., 2019), we also aimed to explore its genetic relations to other G1 isolates from Europe for which sequences are available. As for this purpose, sequencing a significantly larger portion of mtDNA is recommended (e.g. Kinkar *et al*., 2016, 2018*b*, 2018*c*; Laurimäe *et al*., 2018*b*; Ohiolei *et al*., 2020); we aimed here to sequence 11 682 bp of mtDNA as performed for G1 and G3 isolates (Kinkar *et al*., 2018*b*, 2018*c*). However, the isolate from Iceland did yield somewhat shorter high-quality sequence (8141 bp).

**Figure 1.**
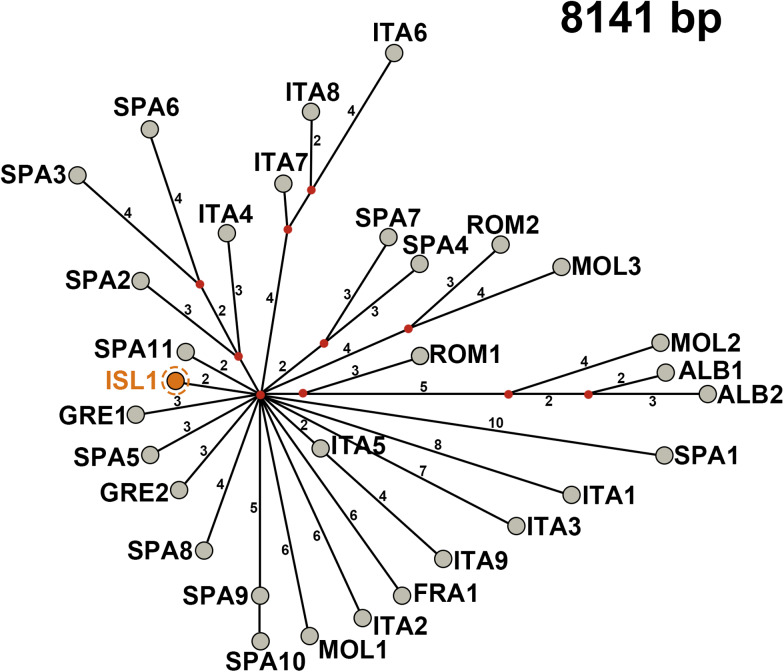
Median-joining network based on 8141 bp of mitochondrial genome sequences of *Echinococcus granulosus sensu stricto* G1 isolate from Iceland and across Europe. The numbers on the lines represent the number of nucleotide differences (mutational steps). Red dots are median vectors (haplotypes not sampled or extinct). The G1 haplotype from Iceland (ISL1) is in orange, surrounded by a dashed circle. Other haplotypes and their countries of origin are designated as 3 letter abbreviations (ALB, Albania; FRA, France; GRE, Greece; ITA, Italy; MOL, Moldova; ROM, Romania; SPA, Spain).

The Icelandic sample sequenced in the present study was genetically relatively similar to the other more ‘contemporary’ G1 samples (Fig. 1). It is likely that the ancestral genetic variant of G1 was introduced together with one of the past populations of sheep to Europe. Previously, Chessa *et al*. (2009) have demonstrated, using genome integrated retroviral sequences (retrotypes), that sheep were dispersed over Europe by 2 different migration waves. During the first wave, sheep with more primitive characteristics (e.g. the Mouflon, Orkney, Soay and the Nordic short-tailed sheep) were confined to the periphery of northern Europe. The second wave involved sheep with improved production traits and these animals have shaped the majority of present-day breeds. Sheep from Iceland are part of the Nordic short-tailed sheep (Aðalsteinsson, 1981), displaying retrotypes similar to those of Faroe Island and Fennoscandian populations (Chessa *et al*., 2009).

### Control efforts of CE in Iceland and elsewhere

As a measure to prevent the reintroduction of *E. granulosus s.l.*, the import of dogs to Iceland was restricted during 1909–1989. The ban was lifted in 1989, with the requirements of an enforced period of quarantine and of specific medical control and anticestodal treatment. At present, in 2023, each dog must be treated twice abroad with approved veterinary medicinal product(s) that has been registered for tapeworms; first at 3–4 weeks, then again 5–10 days prior to the import to Iceland. After arrival in Iceland, at the onset of a 2-week long stay in quarantine, a fecal sample must be examined for the presence of helminth parasites. Three days before leaving the quarantine station, and being handed over to the owner, the dog is treated for the third time with an approved anticestodal drug (Reglugerd ANR 201/2020). Further measures to prevent the re-emergence of CE in Iceland include a yearly, mandatory treatment of all dogs in Iceland with an approved anticestodal drug. Also, by slaughtering ruminants in abattoirs, hydatids are systematically searched for by veterinarians.

Although CE has been diagnosed in humans in other countries in the circumpolar region (e.g. Fennoscandia, Canada, Alaska and northern Russia), these continental regions represent somewhat different ‘settings’ in terms of CE transmission. To date, only island-based CE control programmes, such as in Iceland, have been successful in the eradication of CE transmission, whereas control in a continental setting has proved to be much more challenging (Craig *et al*., 2017). The predominant genotypes and species in continental circumpolar regions are *E. canadensis* genotypes G8 and G10, although surveillance data on CE is incomplete throughout the territories (Davidson *et al*., 2016). In these areas, a synanthropic cycle involving semi-domesticated reindeer and dogs has caused hyperendemicity of CE in several arctic regions in Eurasia, including Fennoscandia and Alaska (Rausch, 2003). In both areas, the synanthropic cycle of CE has been largely eliminated owing to a major change in reindeer husbandry, namely by making herding dogs largely redundant by replacing them with snowmobiles (Rausch, 2003; Oksanen and Lavikainen, 2015).

Eradication or significant reduction of CE is more difficult to accomplish in large continental areas than in islands. However, recent signs of progress in the reduction of CE in continental territories are also evident. For example, in a highly endemic region of Campania in southern Italy, a multidisciplinary One Health approach to combat CE has been implemented since 2010. This involved a complex of procedures and tools, such as surveillance, diagnosis, anthelminthic treatment and public education, which has resulted in significant reduction of CE in livestock (Cringoli *et al*., 2021).

## Conclusion

The current study represents the first molecular characterization for one of the last CE cases recorded in Iceland, revealing *E. granulosus s.s.* genotype G1 as the causative agent. We conclude that it is likely that *E. granulosus s.s.* G1 was one of the representative genotypes circulating among humans and livestock in Iceland prior to the eradication of CE almost half a century ago, and may partly explain why such a large proportion of humans were infected in Iceland prior to the eradication of CE. Thus, decades-old samples hold significant potential to uncover genetic identities of parasites and other pathogens in the past (see also e.g. Flammer *et al*., 2018; Guellil *et al*., 2022). In the future, it would be interesting to explore the Icelandic isolate on a whole-genome scale, facilitated by the availability of a reference genome for genotype G1 (Korhonen *et al*., 2022).

## Data Availability

The 8141 bp mtDNA sequence of the *E. granulosus s.s.* G1 isolate is deposited in GenBank under accession number OQ269593. The supplementary material for this article (Figs S1–S4) can be found at https://www.cambridge.org/core/journals/parasitology.

## Supplementary material

For supplementary material accompanying this paper visit https://doi.org/10.1017/S0031182023000355. click here to view supplementary material
