# Supplementary material for: Cystic echinococcosis in Iceland: a brief history and genetic analysis of a 46-year-old Echinococcus isolate collected prior to the eradication of this zoonotic disease
Source: Parasitology. 2023 Apr 14;150(7):638–43. doi: 10.1017/S0031182023000355 (PMC10261677; doi:10.1017/S0031182023000355)
Supplement: Supplementary file 1 [file S0031182023000355sup001.pdf]

Supplementary File (Figures S1-S4)

Cystic echinococcosis in Iceland: a brief history and genetic analysis of a 46-year-old *Echinococcus* isolate collected prior to the eradication of this zoonotic disease

Urmas Saarma[1]*, Karl Skirnisson[2], Thorunn Soley Björnsdottir[3], Teivi Laurimäe[1], Liina Kinkar[1]

[1] *Department of Zoology, Institute of Ecology and Earth Sciences, University of Tartu, J. Liivi 2, 50409 Tartu, Estonia*

[2] *Laboratory of Parasitology, Institute for Experimental Pathology, University of Iceland, Keldur, Reykjavik, Iceland*

[3] *PCR Laboratory, Institute for Experimental Pathology, University of Iceland, Keldur, Reykjavik, Iceland*

* Corresponding author: Department of Zoology, Institute of Ecology and Earth Sciences, University of Tartu, J. Liivi 2, 50409 Tartu, Estonia. E-mail: Urmas.Saarma@ut.ee

Fig. S1. A man in Iceland with cystic echinococcosis; late 19th century (photo obtained from Matthías Einarsson/Eiríkur Jónsson).

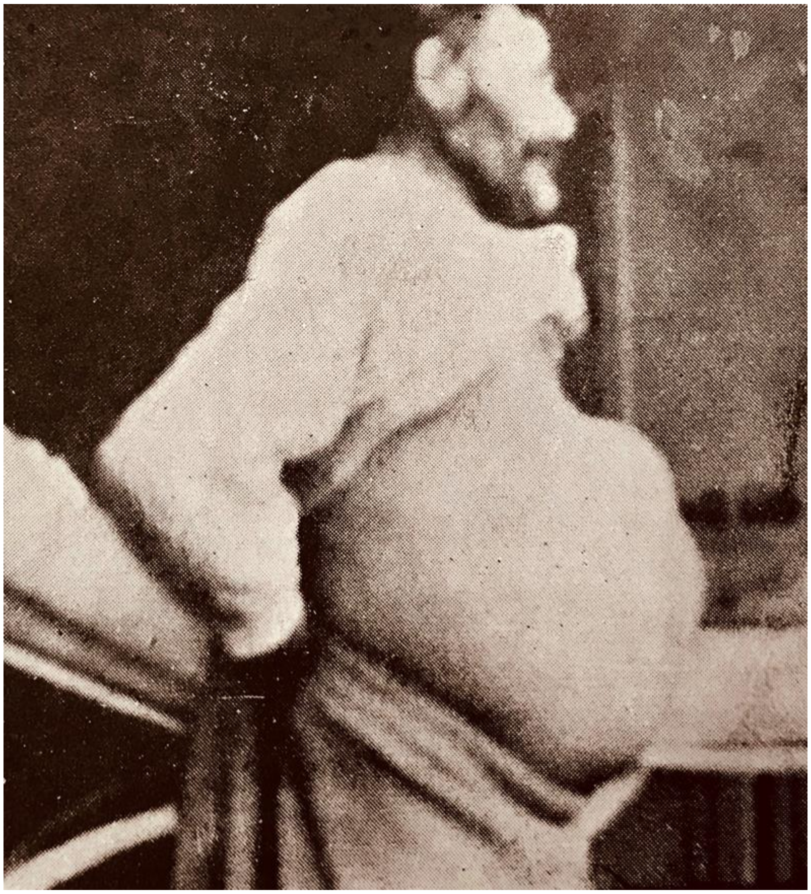

Fig. S2. An *Echinococcus granulosus* cyst surgically removed from a human patient, diameter 43mm (15.2.1984; Iceland). Upper photo: front view; below: view from above.

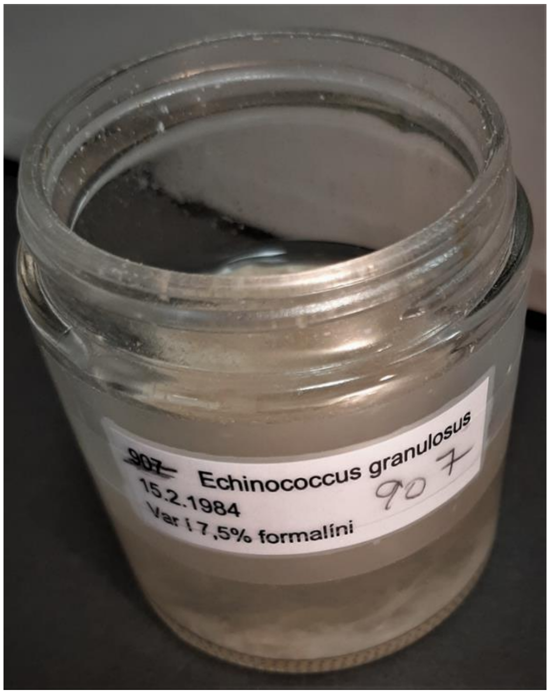

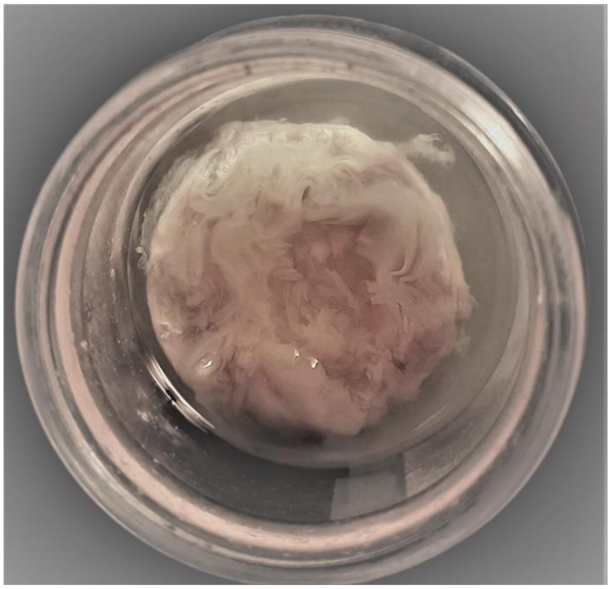

Fig. S3. Microphotograph of the *Echinococcus granulosus* metacestode (larva) isolated from an infected sheep in Iceland (1977). The genotype of this isolate determined in this study was *E. granulosus sensu stricto* G1.

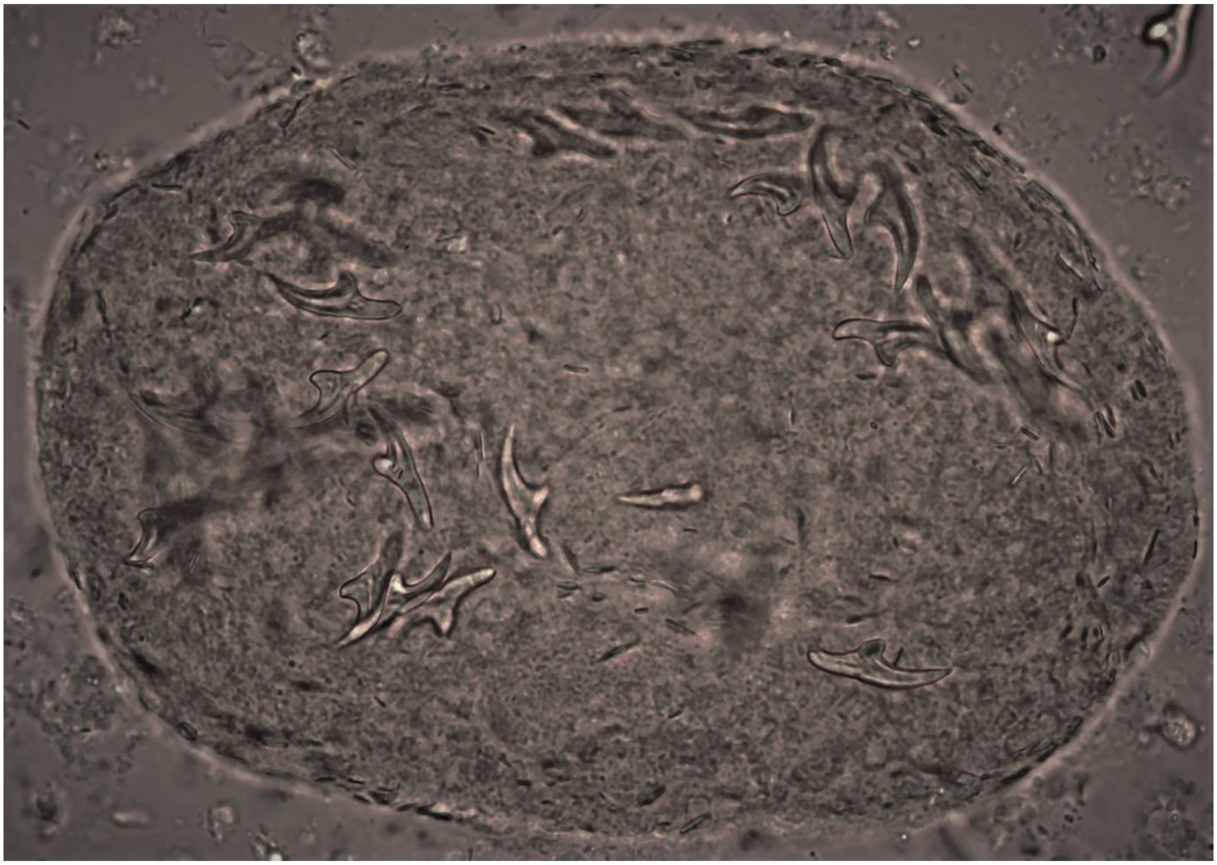

Fig. S4. Examination notes of the sheep infected with *Echinococcus granulosus* taken in 1977 by Halldór Vigfusson (front and back views; in Icelandic).

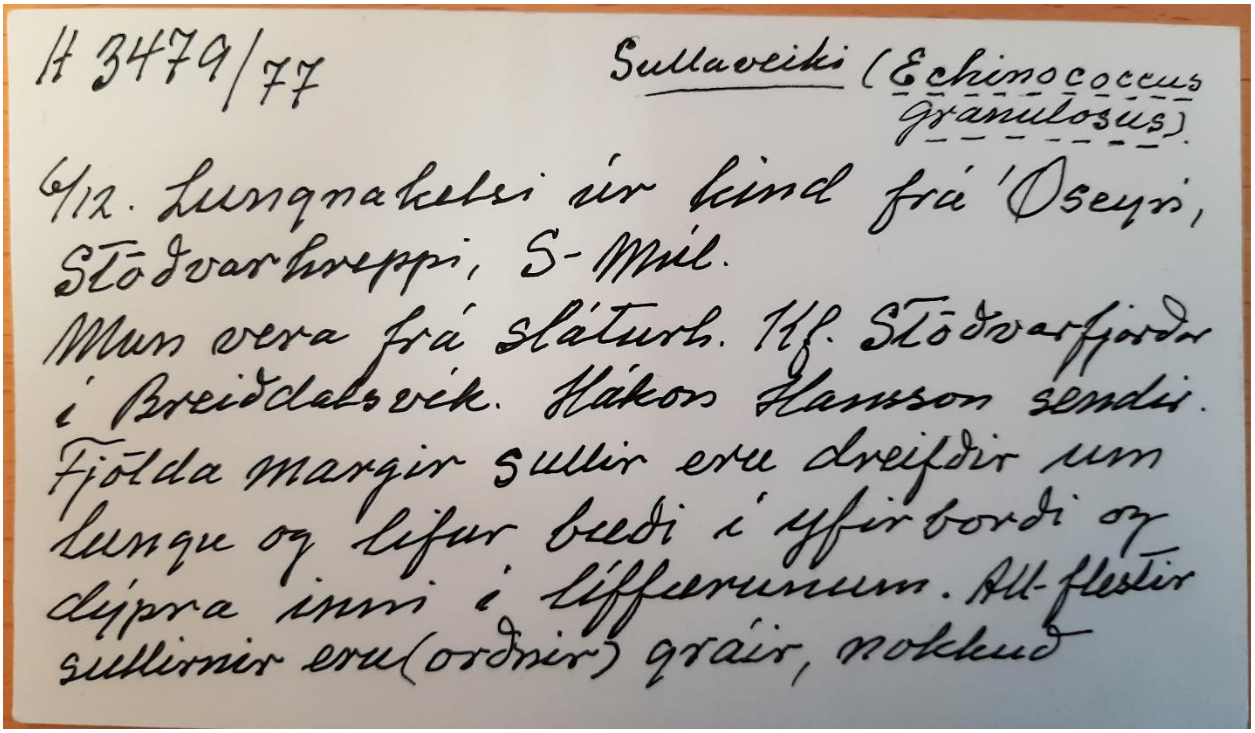

H 3479/77 Sullaveiki (Echinococcus granulosus)

6/12. Lungnakelsi úr kind frá Óseyri, Stöðvarhreppi, S-Múl.
Mun vera frá sláturh. Kf. Stöðvarfjarðar í Breiðdalsvík. Hákon Hansson sendir.
Fjölda margir sullir eru dreifðir um lungu og lifur bæði í yfirborði og dýpra inni í líffærunum. All-flestir sullirnir eru (orðnir) gráir, nokkuð

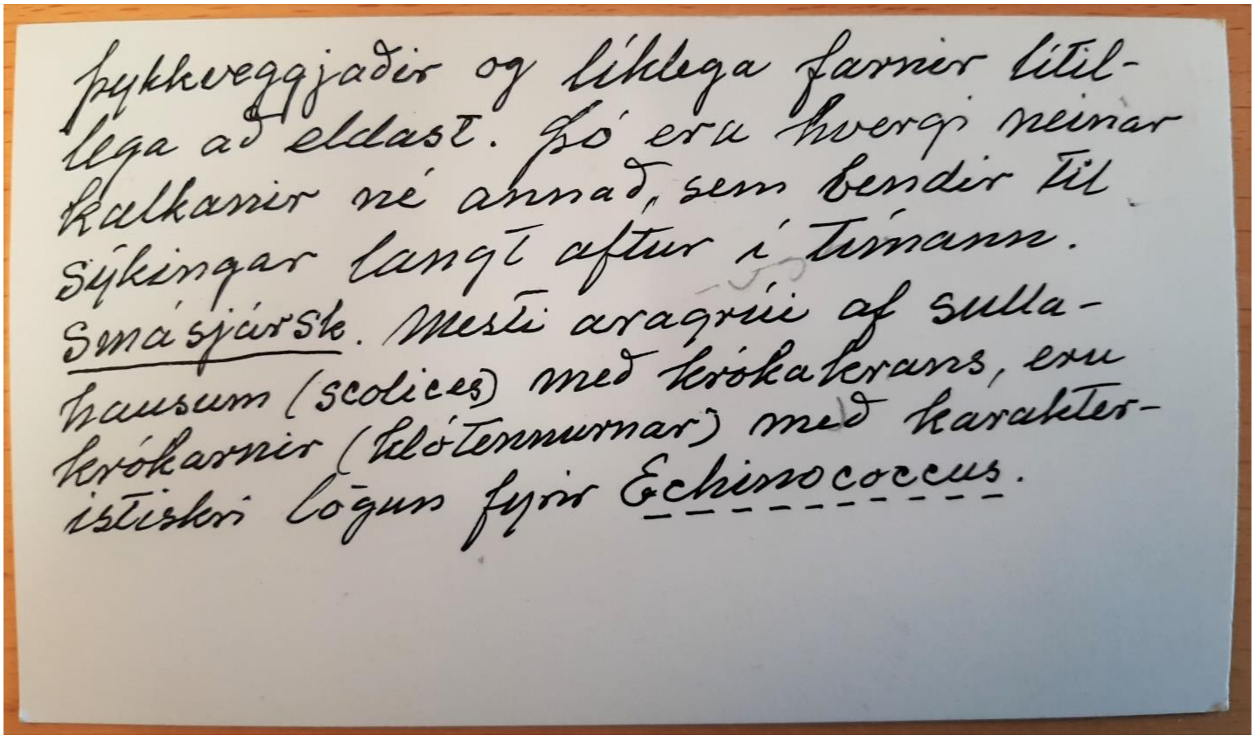

þykkveggjaðir og líklega farnir lítillega að eldast. Þó eru hvergi neinar kalkanir né annað, sem bendir til sýkingar langt aftur í tímann.
Smásjársk. Mesti aragrúi af sulla-hausum (scolices) með krókakrans, eru krókarnir (klótennurnar) með karakter-istiskri lögun fyrir Echinococcus.
